# Doxapram Use for Apnoea of Prematurity in Neonatal Intensive Care

**DOI:** 10.1155/2013/251047

**Published:** 2013-11-26

**Authors:** S. A. Prins, S. J. A. Pans, M. M. van Weissenbruch, F. J. Walther, S. H. P. Simons

**Affiliations:** ^1^Division of Neonatology, Department of Pediatrics, Leiden University Medical Center, J6-S, Albinusdreef 2, 2333 ZA Leiden, The Netherlands; ^2^Division of Neonatology, Department of Pediatrics, VU University Medical Center, Amsterdam, The Netherlands; ^3^Division of Neonatology, Department of Pediatrics, Erasmus MC Sophia Children's Hospital, Rotterdam, The Netherlands

## Abstract

Apnoea of prematurity is treated with noninvasive respiratory therapy and methylxanthines. For therapy unresponsive apnoea doxapram is often prescibed in preterm neonates. The duration, dosage and route of administration of doxapram together with its efficacy was evaluated in two Dutch neonatal intensive care. Outcome concerning short-term safety and neonatal morbidity were evaluated. During 5 years, 122 of 1,501 admitted newborns <32 weeks of gestational age received doxapram. 64.8% of patients did not need intubation after doxapram. 25% of treated neonates were <27 weeks of gestation. A positive response to doxapram therapy on apnoea was associated with longer duration of doxapram usage (*P* < 0.001), lower mean doses (*P* < 0.003), and less days of intensive care (median 33 versus 42 days; *P* < 0.002). No patients died during doxapram therapy. Incidence of necrotizing enterocolitis, intraventricular hemorrhage, periventricular leukomalacia, retinopathy of prematurity, persistent ductus arteriosus, or worsening of pulmonary condition did not increase during doxapram therapy. Doxapram is frequently used for apnoea of prematurity, despite a lack of data on short-term efficacy and long-term safety. Until efficacy and safety are confirmed in prospective trials, doxapram should be used with caution.

## 1. Introduction

Recent advances in obstetrical and neonatal intensive care management have increased the survival rates of very low birth weight infants. In these infants, artificial ventilation is related to potential iatrogenic lung damage and therefore reduced to a minimum[1]. The introduction of new strategies of surfactant therapy includes a very short period of mechanical ventilation (InSurE) [2] or even avoidance of endotracheal intubation [3] in newborns with respiratory distress syndrome. As a consequence, noninvasive respiratory therapy has become increasingly important and is used in even the youngest neonates. In these infants, apnoea has emerged as a major clinical problem, manifested by an unstable respiratory rhythm reflecting the immaturity of the respiratory control systems. Apnoea appears to be harmful to the brain when associated with significant hypoxemia [4]. Methylxanthines, such as theophylline and caffeine, are the mainstay pharmacological treatment for apnoea and have proven to reduce chronic lung disease and long-term outcome [5, 6]. 

In line with current international consensus in the 2 reporting NICUs, caffeine base is given with a loading dose of 10 mg/kg and a maintenance dose of 5 mg/kg/day. Although Steer et al. and Gray et al. published very reassuring data [7, 8] on short-and long-term outcome of high dose caffeine, more data are needed to change the existing caffeine dosing protocol.

If apnoea persists under methylxanthines, doxapram may be considered as the therapy of last resort before endotracheal intubation and mechanical ventilation.

Doxapram is a respiratory stimulant that encourages breathing by stimulating both peripheral and central chemoreceptors [9–11]. Doxapram was introduced more than 25 years ago into neonatology to treat neonates struggling with persistent idiopathic apnoea of prematurity [12, 13]. Pharmacokinetics and routes of administration were explored [14–16]. Some randomized controlled trials studied doxapram, but none were placebo controlled [17–19]. A number of observational trials suggested potential short-and long-term side effects such as hypertension and irritability. The most devastating, however, were the suggested negative effects of doxapram on cerebral oxygenation and long-term mental development [20–22]. 

These concerns about long-term outcome and important improvements of neonatal care such as the introduction of surfactant therapy and maternal corticosteroids pushed the use of doxapram in preterm infants to the background.

Nevertheless, we recently noticed a reintroduction of doxapram use in our departments in premature infants with superficial breathing patterns and apnoeas that are unresponsive to methylxanthines. In this situation of persistent apnoea and imminent neonatal respiratory insufficiency, doxapram is used as rescue medication. Doxapram is administered in order to prevent preterm neonates from endotracheal intubation and mechanical ventilation. However, doxapram is still off-label in premature infants and evidence about efficacy and safety is lacking [23]. We therefore performed an audit on doxapram use and the effects of doxapram use in two Dutch tertiary NICUs during the last 5 years.

## 2. Methods

### 2.1. Ethics Statement

Due to the retrospective nature of our audit, the Committee of Medical Ethics of LUMC and the Medical Ethical Committee of VU University Medical Center have declared that formal approval was not required.

### 2.2. Data Collection

Data of patients who received doxapram were analyzed from two tertiary Neonatal Intensive Care Units (Leiden University Medical Center, Leiden, center 1; VU University Medical Center Amsterdam, center 2) in the Netherlands. From prospectively collected drug databases all eligible patients from 2006 till 2011 were selected in both centers. Patient's medical records including drug charts were analyzed to obtain all necessary data. Data on total numbers of patients admitted per gestational age group in both centers were also collected. 

### 2.3. Caffeine Treatment Policies

In both centers, neonates were treated with caffeine base formulations. A loading dose of 10 mg/kg was given, with daily subsequent doses of 5 mg/kg intravenously or orally. In center 1, caffeine was occasionally switched to theophylline with a dose of 6–8 mg/kg/day. Although promising data regarding higher daily caffeine doses were published in 2004 and 2011, both NICUs chose to use standard dosing regimens [7, 8].

### 2.4. Doxapram Treatment Policies

The doxapram treatment policies of both centers were different during the study period. In center 1, intravenous and oral doxapram were used until 2011 and dosed based on personal experience of the attending physician. During doxapram treatment, caffeine was often replaced by theophylline. In center 2, doxapram was incorporated into a written apnoea policy from 2010. Before 2010, it was used and dosed based on personal experience of the attending physician. The written policy advised intravenous use of doxapram with a starting dose of 0.5 mg/kg/hr that could be increased every 12 hours up to 2.0 mg/kg/hr. The protocol advised to consider the use of doxapram after persistent apnoea with maximal noninvasive ventilatory support and caffeine therapy.

All patients who received doxapram were included into the study. Data about pregnancy, delivery, and baseline patient characteristics were analysed in order to determine which group of patients received doxapram.

The use of doxapram was evaluated by its duration, dosage, route of administration and efficacy. The average dose of doxapram for each patient was calculated. Doxapram therapy was defined as successful if no endotracheal intubation due to apnoea was necessary. No criteria for respiratory insufficiency or endotracheal intubation were included in the protocols of both centers. Attending physicians were allowed to decide if endotracheal intubation was necessary. 

Outcome of patients was analyzed by collecting data about mortality, short-term morbidity (incidence of necrotizing enterocolitis, intraventricular hemorrhage, periventricular leukomalacia, retinopathy of prematurity, and persistent ductus arteriosus) and pulmonary condition.

Data were analyzed using SPSS 15.0. Background characteristics; doxapram data and morbidity of patients who were successfully and unsuccessfully treated with doxapram were compared using Chi-square and Fisher's exact tests as well as non-parametric Mann-Whitney *U* statistical tests wherever appropriate.

## 3. Results

From 2005 to 2010, 122 out of 1,501 admitted premature infants with a gestational age less than 32 weeks in the two centers received doxapram (8.13%). Background characteristics of the patients are shown in Table 1. All neonates received caffeine with a loading dose of 10 mg/kg and daily maintenance doses of 5 mg/kg. Until 2010, all patients in center 1 receiving doxapram were switched to theophylline therapy.

Doxapram was most often used in the youngest neonates with 26.7%, 21.4%, and 26.6% of all preterms born after 24, 25, and 26 weeks of gestation, respectively. Doxapram was more frequently used in center 1 (84/802; 10.5%) than in center 2 (38/699; 5.4%). There was a wide variation in the doxapram doses used and in the duration of therapy (Table 2). Median postconceptional age at the start of doxapram therapy was 27.3 weeks with the youngest infant being 24 4/7 weeks and the oldest being 31 3/7 weeks at the start of doxapram therapy. Doxapram was initiated at a median postnatal age of 13.5 days (IQR 11 days) and patients received doxapram for a median duration of 108 hrs (IQR 147 hours). Nine patients (7.4%) received a doxapram loading dose (median 2.5 mg/kg). In all other patients, a continuous dose was started orally or intravenously without a loading dose. The cumulative dose of doxapram ranged from 0.33 mg to 781.5 mg doxapram per kg bodyweight.

Twenty-nine newborns (24%) received 2 courses of doxapram; 2 patients (1,6%) received a third course of doxapram therapy.

Outcomes of the patients who received doxapram are shown in Table 3. In 6 cases, it could not be determined if doxapram was successful, as it was unclear if the patient was intubated because of persistent apnoea or because of other reasons, such as infection. In 79 out of 116 neonates (64.8%), no intubation and endotracheal ventilation was necessary to treat apnoea. Doxapram was considered to be successful in these cases. Baseline characteristics were comparable between patients in whom doxapram was successful compared to those with unsuccessful treatment (Table 1). If doxapram treatment was successful, it was used longer (Median (IQR): 5.5 (5.7) days versus 1 (4.65) day; Mann-Whitney *U* 722, *P* < 0.001) but in lower average dosages (Median (IQR): 0.97 (0.62) mg/kg/hr versus 1.38 (0.94), Mann-Whitney *U* 952, *P* < 0.003). Patients who received doxapram therapy for a long period of time, incidence of necrotizing enterocolitis, intraventricular hemorrhage, periventricular leukomalacia, retinopathy of prematurity, persistent ductus arteriosus, or worsening of pulmonary condition did not increase. Their stay at the neonatal intensive care unit was shorter (median (IQR): 33 (26) versus 42 (34) days; Mann-Whitney *U* 815, *P* = 0.002). 

Off all the included patients, 49.2% received doxapram intravenously, 41.5% received doxapram orally and 9.3% received doxapram via both routes. If intravenous doxapram was effective and the neonates did not need an intravenous route anymore, doxapram was given through the nasogastric tube in center 1 only. Doxapram therapy was successful in 67% of intravenously treated and 70% of orally treated neonates. If both routes were used subsequently, the success rate was 55% (Chi-Square test: *P* = 0.63).

## 4. Discussion

In this study, we show that doxapram is frequently used in preterm newborns. Up to 25 percent of the youngest premature infants received doxapram in the NICU. Almost two third of all treated newborns did not progress to ventilatory support although they were suffering from frequent apnoea before treatment with doxapram.

These data suggest a potential important role for doxapram in the treatment of apnoea of prematurity. However, it is remarkable that underlying evidence about the efficacy and safety from well-designed clinical trials is almost completely lacking.

For 50 years, doxapram has been used as an emergency agent in adults suffering from life-threatening respiratory insufficiency [10]. It was the anesthesiologist Gupta who first reported the successful administration of doxapram in newborns with breathing difficulties after birth [9] and from the 1980s on doxapram became popular for the treatment of neonates struggling with persistent idiopathic apnoea of prematurity [12, 13]. In the past 30 years, only three randomized controlled trials compared doxapram with other therapeutic strategies in a total of 86 neonates. Peliowski and Finer compared doxapram with theophylline and placebo in a crossover design in 31 preterm newborns with apnoea [19]. They found a short-term doxapram treatment success rate of 64 percent in neonates with a mean gestation of 31 weeks. Eyal et al. found success rates of, respectively, 53 and 55 percent comparing doxapram monotherapy with aminophylline monotherapy [17]. If doxapram was used in addition to aminophylline, it was successful in 8 out of 10 patients compared to 0 out of 9 placebo treated newborns. Huon et al. studied the effects of doxapram after extubation and found a reintubation rate of 21% compared to 47% of placebo treated newborns [18]. In analogy with our data, they suggested that doxapram is successful in treating apnoea of prematurity in at least a considerable part of the premature neonates. A wide variation in used doxapram dosages varying from 0.18 up to 4 milligram per kg bodyweight per hour was found in the current study. As suggested by Barrington et al., most patients started with a dose of 0.5 mg/kg/hr [14]. The same study also showed increasing success rates of doxapram with increasing dosages up to 2.5 mg/kg/hr. A dose effect relationship might be apparent but has until now not been studied sufficiently.

A prospective doxapram dose finding study and randomised placebo controlled analysis of the effectiveness of doxapram with a sufficient number of patients is needed to determine the actual efficacy and effective dosages of doxapram on apnoea in premature newborns.

As this was not a randomized controlled trial, hard conclusions cannot be drawn. However, retrospectively, we found positive results in almost 65% of infants treated with doxapram. Response to doxapram therapy seemed to be associated with a shorter stay in the neonatal intensive care unit without serious side effects. Protecting newborns from reintubation and ventilation might theoretically protect them against bronchopulmonary dysplasia but the doxapram responders merely depict the neonates who initially had less severe lung problems. The design of the current study precludes any conclusion about a causal relationship.

The most important and potentially dangerous suggested side effect consists of decreased cerebral oxygenation and blood flow velocity [20]. This might result in decreased cerebral perfusion and damage to the developing brain leading to long-term developmental delay [20, 21]. As our study was not a randomised controlled trial, no control group is available. Doxapram is given to a specific category of preterm neonates with pulmonary problems, therefore no representative control could be found in the rest of the patients on our wards during the study period. Routine follow-up of the doxapram treated premature infants has not shown long-term safety issues at this time. The long-term follow-up is absolutely necessary in a future RCT.

Our audit merely shows that doxapram has been reintroduced into Dutch NICUs. The reintroduction of doxapram can be explained by the introduction of new techniques aimed at minimizing invasive ventilation of premature neonates. Techniques such as InSurE,CPAP, nasal intermittent mandatory ventilation, and surfactant without intubation may ultimately lead to an increased use of doxapram as pointed out by Kribs et al. [3]. Surfactant administration through a nasogastric tube resulted in doxapram use in 28 percent of the surfactant treated neonates via a nasogastric tube compared to 17 percent of patients receiving standard surfactant therapy [3]. In the coming years, a significant further increase of doxapram administration can be expected. 

Therefore, this study shows that doxapram as off-label drug is frequently used in premature neonates in neonatal nurseries and especially in the youngest infants. As increased use of doxapram is to be expected, prospective well-designed studies are needed to address the issues of short- and long-term safety. Until then, doxapram should be used with caution. 

## Figures and Tables

**Table 1:** Patient characteristics of included premature neonates who received doxapram (Total *N* = 122).

	Overall (*N* = 122)		Successfully treated patients (*N* = 79)		Unsuccessfully treated patients (*N* = 37)		*P*
	Percentage	Median (IQR)	Percentage	Median (IQR)	Percentage	Median (IQR)	
Birth weight (gram)		937 (267)		980 (267)		915 (332)	0.21
Gestational age (weeks)		27 (1.7)		27 (2.2)		27 (2.6)	0.21
Apgar score							
1 min		5 (4)		6 (4)		5 (4)	0.09
5 min		8 (2)		8 (2)		8 (2)	0.08
Mode of delivery:							
Vaginal	66.7%		68.0%		61.1%		
Caesarean section	33.3%		32.0%		38.9%		
Maternal corticosteroids							
Adequate*	42.1%		43.2%		37.1%		
Inadequate	31.6%		32.4%		31.4%		
Centre1/centre 2	68.9%/31.1%		69.2%/65.8%		30.8%/34.2%		
Gender (m/f)	50%/50%		49.4%/50.6%		54.1/45.9%		
Twin	35.2%		41.7%		24.3%		
Primary ventilation	37.7%		27.8%		56.8%		
Ventilation before doxapram	80.7%		76.3%		86.5%		
Surfactant therapy	74.6%		67.1%		86.1%		
Caffeine	98.3%		98.7%		97.3%		
Theophylline	32.0%		31.6%		29.7%		

*Maternal corticosteroid therapy was judged adequate if two doses were given more than 24 hours before birth.

**Table 2:** Doxapram data.

	*N* (%)	Median (IQR) (min–max)
Success rate of doxapram	79/116 (64.8%)	
Route of administration (IV/oral/both)	58 (49.2)/49 (41.5)/11 (9.3)	
Number of doxapram episodes (1/2/3)	92 (75.4)/29 (23.8)/1 (0.8)	
Postnatal age (days)		13.5 (11) (1–40)
Total duration of doxapram use (hrs)		108 (147) (0.5–698)
Average dose of doxapram (mg/kg/hour)		1.07 (0.66) (0.32–5.44)
Cumulative dose doxapram		116 (186) (0.3–859.6)
Cumulative dose doxapram (per kg)		109.5 (174.2) (0.33–781.5)

**Table 3:** Short-term outcome of the patients who received doxapram.

	Total		Successfully treated patients (*N* = 79)		Unsuccessfully treated patients (*N* = 37)		P*
	*N* (%)	Median (IQR) (min–max)	%	Median (IQR)	%	Median (IQR)	
Neonatal survival^#^	117/122 (95.9)		**1/79 (98.7)**		**4/37 (89.2)**		**0.035**
Number of ventilatory days		7 (12) (0–72)		5 (12)		11 (13)	**<0.001**
Number of NICU days		38 (31) (6–120)		33 (26)		42 (34)	**0.002**
Pneumothorax	6/122 (4.9)		3/76 (3.9)		2/37 (5.4)		0.65
Corticosteroids for detubation	27/122 (22.1)		11/79 (13.9)		13/37 (35.1)		**0.013**
NEC	27/122 (22.1)		15/79 (19.0)		10/37 (27.0)		0.34
IVH (all grades)	40/122 (32.8)		23/79 (29.1)		16/37 (43.2)		0.15
PVL (all grades)	35/120 (29.2)		19/78 (24.4)		14/36 (38.9)		0.13
Persistent ductus arteriosus	44/122 (36.1)		26/79 (32.9)		14/37 (37.8)		0.68
ROP	17/112 (15.2)		8/72 (11.1)		7/34 (20.6)		0.24

*Mann-Whitney *U* test and Fisher's Exact test.

^#^No patients died during doxapram treatment.

## References

[1] Kinsella JP, Greenough A, Abman SH (2006). Bronchopulmonary dysplasia. *The Lancet*.

[2] Engle WA, Stark AR, Adamkin DH (2008). Surfactant-replacement therapy for respiratory distress in the preterm and term neonate. *Pediatrics*.

[3] Kribs A, Härtel C, Kattner E (2010). Surfactant without intubation in preterm infants with respiratory distress: first multi-center data. *Klinische Padiatrie*.

[4] Pillekamp F, Hermann C, Keller T, von Gontard A, Kribs A, Roth B (2007). Factors influencing apnea and bradycardia of prematurity—implications for neurodevelopment. *Neonatology*.

[5] Schmidt B, Roberts RS, Davis P (2007). Long-term effects of caffeine therapy for apnea of prematurity. *The New England Journal of Medicine*.

[6] Schmidt B, Anderson PJ, Doyle LW (2012). Survival without disability to age 5 years after neonatal caffeine therapy for apnea of prematurity. *Journal of the American Medical Association*.

[7] Steer P, Flenady V, Shearman A (2004). High dose caffeine citrate for extubation of preterm infants: a randomised controlled trial. *Archives of Disease in Childhood*.

[8] Gray PH, Flenady VJ, Charles BG, Steer PA (2011). Caffeine citrate for very preterm infants: effects on development, temperament and behaviour. *Journal of Paediatrics and Child Health*.

[9] Gupta PK, Moore J, Lewis MA, Dundee JW (1972). Clinical trial of doxapram hydrochloride in the resuscitation of the newborn. *British Journal of Anaesthesia*.

[10] Siker ES, Mustafa K, Wolfson B (1964). The analeptic effects of doxapram hydrochloride on thiopentone induced depression. *British Journal of Anaesthesia*.

[11] Yost CS (2006). A new look at the respiratory stimulant doxapram. *CNS Drug Reviews*.

[12] Alpan G, Eyal F, Sagi E (1984). Doxapram in the treatment of idiopathic apnea of prematurity unresponsive to aminophylline. *Journal of Pediatrics*.

[13] Sagi E, Eyal F, Alpan G (1984). Idiopathic apnoea of prematurity treated with doxapram and aminophylline. *Archives of Disease in Childhood*.

[14] Barrington KJ, Finer NN, Torok-Both G (1987). Dose-response relationship of doxapram in the therapy for refractory idiopathic apnea of prematurity. *Pediatrics*.

[15] Hayakawa F, Hakamada S, Kuno K, Nakashima T, Miyachi Y (1986). Doxapram in the treatment of idiopathic apnea of prematurity: desirable dosage and serum concentrations. *Journal of Pediatrics*.

[16] Bairam A, Akramoff-Gershan L, Beharry K, Laudignon N, Papageorgiou A, Aranda JV (1991). Gastrointestinal absorption of doxapram in neonates. *American Journal of Perinatology*.

[17] Eyal F, Alpan G, Sagi E (1985). Aminophylline versus doxapram in idiopathic apnea of prematurity: a double-blind controlled study. *Pediatrics*.

[18] Huon C, Rey E, Mussat P, Parat S, Moriette G (1998). Low-dose doxapram for treatment of apnoea following early weaning in very low birthweight infants: a randomized, double-blind study. *Acta Paediatrica*.

[19] Peliowski A, Finer NN (1990). A blinded, randomized, placebo-controlled trial to compare theophylline and doxapram for the treatment of apnea of prematurity. *Journal of Pediatrics*.

[20] Dani C, Bertini G, Pezzati M (2006). Brain hemodynamic effects of doxapram in preterm infants. *Biology of the Neonate*.

[21] Lando A, Klamer A, Jonsbo F, Weiss J, Greisen G (2005). Doxapram and developmental delay at 12 months in children born extremely preterm. *Acta Paediatrica*.

[22] Sreenan C, Etches PC, Demianczuk N, Robertson CMT (2001). Isolated mental developmental delay in very low birth weight infants: association with prolonged doxapram therapy for apnea. *Journal of Pediatrics*.

[23] Henderson-Smart DJ, Davis PG (2000). Prophylactic doxapram for the prevention of morbidity and mortality in preterm infants undergoing endotracheal extubation. *Cochrane Database of Systematic Reviews*.

